# Food, fizzy, and football: promoting unhealthy food and beverages through sport - a New Zealand case study

**DOI:** 10.1186/1471-2458-13-126

**Published:** 2013-02-11

**Authors:** Mary-Ann Carter, Louise Signal, Richard Edwards, Janet Hoek, Anthony Maher

**Affiliations:** 1 Department of Public Health, University of Otago, 23a Mein St Wellington, Wellington, New Zealand; 2 Department of Marketing, University of Otago, PO Box 56, Dunedin, New Zealand; 3 Auckland District Health Board, Private Bag 92024, Auckland Mail Centre, Auckland, New Zealand

**Keywords:** Sport, Sponsorship, Food, Beverage, Marketing

## Abstract

**Background:**

High participation rates in sport and increasing recognition of how diet benefits athletic performance suggest sports settings may be ideal locations for promoting healthy eating. While research has demonstrated the effect of tobacco and alcohol sponsorship on consumption, particularly among youth, few studies have examined the extent or impact of food and beverage company sponsorship in sport. Studies using brand logos as a measure suggest unhealthy foods and beverages dominate sports sponsorship. However, as marketing goes beyond the use of brand livery, research examining how marketers support sponsorships that create brand associations encouraging consumer purchase is also required. This study aimed to identify the characteristics and extent of sponsorships and associated marketing by food and non-alcoholic beverage brands and companies through a case study of New Zealand sport.

**Methods:**

We conducted a systematic review of 308 websites of national and regional New Zealand sporting organisations to identify food and beverage sponsors, which were then classified as healthy or unhealthy using nutrient criteria for energy, fat, sodium and fibre levels. We interviewed 18 key informants from national and regional sporting organisations about sponsorships.

**Results:**

Food and beverage sponsorship of sport is not extensive in New Zealand. However, both healthy and unhealthy brands and companies do sponsor sport. Relatively few support their sponsorships with additional marketing. Interviews revealed that although many sports organisations felt concerned about associating themselves with unhealthy foods or beverages, others considered sponsorship income more important.

**Conclusions:**

While there is limited food and beverage sponsorship of New Zealand sport, unhealthy food and beverage brands and companies do sponsor sport. The few that use additional marketing activities create repeat exposure for their brands, many of which target children. The findings suggest policies that restrict sponsorship of sports by unhealthy food and beverage manufacturers may help limit children’s exposure to unhealthy food marketing within New Zealand sports settings. Given the global nature of the food industry, the findings of this New Zealand case study may be relevant elsewhere.

## Background

High participation rates in formal sport and consistent pairing of recommendations linking physical activity and healthy eating [1-3] suggest sports settings may be ideal locations for encouraging improved nutrition. Studies in Australia have demonstrated how health sponsorship funding can create healthier environments in sports clubs [4-6].

Although elite sports people understand the role diet plays in enhancing their performance [7], the sponsorship relationships between sporting organisations and food and beverage brands and companies do not always reinforce either sports-related or more general nutrition recommendations. For example, McDonald’s sponsorship of the Olympic Games associates consumption of energy-dense, nutrient-poor food with high performance sport. This pairing suggests that elite sports people consume and endorse these foods and that consumption of energy-dense nutrient-poor foods is consistent with outstanding sporting performance. This message clearly challenges and undermines nutrition advice from health and dietary professionals.

Little is known about food and beverage marketing or the food environment in sports settings [8], and few studies have investigated the characteristics and extent of food and beverage company sponsorship in sport. A survey of children’s sports clubs found 17 percent of all sponsors were food or beverage companies and half of these did not meet criteria classifying them as healthy [9]. A recent Australian study audited websites of national and state sporting organisations of popular sports for children and found that nine percent (n=38) of sponsors were food companies. Sixty-three percent of these food companies did not meet criteria classifying them as healthy [10]. A New Zealand (NZ) study assessing club, national, and regional sporting organisations’ websites for evidence of sponsorship found more unhealthy food sponsorship in junior sport when compared to all other types of sponsorship [11]. Both studies used the presence of a sponsor’s logo as a measure of sponsorship. However neither describes *how* marketers leverage sponsorship with other marketing activities.

Behaviour modification theory suggests repeatedly linking a product with an external stimuli (e.g. a sports match) and advertising increases the probability of product purchase [12]. Sponsorships maintain brand salience (ensuring consumers notice and remember the brand), reinforce purchase behaviour, and remind consumers of brands’ favourable attributes [13]. Sponsorships link brands with associations of excellence and sporting prowess, and suggest purchasing and consuming the brand is a conduit to these attributes. Sporting heroes thus act as role models and vicarious instructors who educate consumers about brands and imply that purchasing them will enable consumers to access desirable and aspirational characteristics [12,14].

To maximise benefits delivered by sponsorship, many marketers augment investment in teams, individuals, or events with additional advertising and marketing support. These additional activities enhance the brand associations created through sponsorship and extend the reach achieved [15,16]. Some studies suggest frequent exposure to advertising that supports sponsorship reminds consumers of brand associations and increases brand recall, purchase intentions, and actual purchase [17,18]. Studies into alcohol and tobacco sponsorship illustrate how brand associations and vicarious learning promote, stimulate, and reinforce behaviour. Sports sponsorship by tobacco companies increased children’s brand recognition and experimentation with smoking [19-21]. Similarly sponsorship enhanced adolescent boys’ intentions to drink alcohol, and created positive attitudes towards alcohol in girls [22].

NZers are passionate about sport; in any week 79% of adults and most children and young people participate in at least one sport or recreation activity [23,24]. Popular sports attract large television audiences with rugby dominating NZ television ratings during 2011 [25]. However, despite NZers’ love of sport, sporting organisations face increasing financial pressure; at the same time, competition to attract sponsorship income has intensified [26]. As sport sponsorship has grown so too has the use of integrated marketing communications (IMC), which join sponsorship with other marketing activities to gain synergistic benefits [27]. At present we know little about the extent of integrated promotions to enhance sponsorship’s reach and influence. This study aimed to identify the characteristics and extent of sponsorships and associated marketing by food and non-alcoholic beverage companies through a case study of NZ sport.

## Methods

### Website review

We identified 58 sports played in NZ from Sport and Recreation New Zealand data analysing NZers’ participation in sport [23]. Google searches identified all national and regional sporting organisations’ (NSO/RSOs) websites for all 58 sports (n=308). One author (MC) examined all pages of each identified website between November and December 2010 for evidence of sponsorship. We defined food related sponsors as ‘food or beverage companies or brands whose company or brand logo featured on the NSO/RSO website and which the sporting code formally identified as an official sponsor or partner, or that had naming rights of teams or tournaments. Data collected included brand or company logos (counted if present) and their location on the website e.g. front page, sponsor’s page. Another observer independently examined a sample of 10% (n=30). An Intra Class Correlation Coefficient of 0.92 was calculated, indicating a reasonable level of agreement between researchers [28].

We grouped food and beverage company sponsors into the following categories for analysis: food and beverage brands; food and beverage companies; quick service restaurants; bars or restaurants, and supermarkets. The New Zealand Food and Beverage Classification System (NZF&B) was used to classify individual foods or beverages (brands) and companies [29]. The NZF&B system was developed by independent nutrition experts for the NZ Ministry of Health to classify foods and beverages in school canteens. The NZF&B system was chosen for this study because it considers NZ nutrition guidelines and nutrient composition of NZ foods. Nutrient criteria assess energy, total fat, saturated fat, sodium and fibre with specific criteria for different product groups. Foods in this study classified as healthy met all aspects of each criterion. Foods with nutrient content exceeding any aspect of the criteria were classified as unhealthy. Product lists were obtained from websites of companies identified as sponsors and all products classified using the NZF&B system. Companies were considered healthy when at least half their products met the NZF&B criteria for classification as healthy. Other companies not meeting these criteria were classified as manufacturing unhealthy food products. We classified quick service restaurants (e.g. McDonalds, Burger King, KFC) as unhealthy because of the high proportion of energy-dense foods sold. Bars and restaurants were not classified because the extent and nature of food sold was unclear. Supermarkets were not classified recognising they sell both healthy and unhealthy foods as well as a range of other products. Tea and coffee are not included in the NZF&B system and therefore not classified.

### Key informant interviews

Eighteen key informant interviews were conducted between August and November 2010. We purposely selected NSO/RSO participants to represent a range of sports including those played in summer and winter, by both genders, and by people from a range of ethnic groups. Senior managers from athletics, basketball, baseball, cricket, hockey, netball, football, swimming, tennis, touch rugby, rugby, and rugby league participated in interviews as did managers from key organisations supporting the delivery of all sports. Key organisations included government organisations (Sport New Zealand, the Health Sponsorship Council) and non-government regional sports trusts. Informants were selected for their extensive knowledge of their sport.

The first author (MC) conducted all interviews in person, except two which were by phone. Interviews followed a semi-structured interview guide exploring participants’ perceptions of the food and beverage companies sponsoring sport and the supporting marketing strategies used. Interviews took between 20 minutes and one hour to complete, were taped, and the recordings transcribed. We coded transcripts using the software NVivo8 and one author (MC) conducted a thematic analysis of the interview transcripts to identify, analyse and report patterns within the transcripts [30]. Themes were identified inductively with the importance of the themes identified based on; the relevance of what was said in relation to the overall research question, the extent to which it captured an important aspect of this study, and the number of informants discussing this aspect [30]. We thoroughly read interview transcripts several times to identify themes and subthemes [31]. Two authors (LS, MC) agreed on themes prior to coding. The Ethics Committee, Department of Public Health, University of Otago approved the study.

## Results

### Website review

Websites of 58 national sporting organisations (NSOs) and 250 regional sporting organisations (RSOs) were located and searched for evidence of food or beverage company sponsorship. Results are presented in Table 1. The logos of food or beverage company sponsors appeared 186 times on 74 websites (24% of websites accessed). The logos represented 131 individual food or beverage companies or brands. No logos appeared on 234 websites (76%). Of the websites sponsored by food and beverage companies, nine belonged to NSOs and 65 to RSOs.

**Table 1:** Food and beverage sponsors of national and regional sporting organisations

**Category**	**Number of sponsors**		**Classified healthy**	**Classified unhealthy**	**Unclassified**
	**n**	**(%)**	**n**	**n**	**n**
**Bars/restaurants**	63	(48)	0	0	63
**Food companies**	36	(27.5)	25	10	1
**Brands**	17	(12.9)	4	7	6
**Quick service restaurants**	12	(9.2)	0	12	0
**Supermarkets**	3	(2.4)	0	0	3
**Total**	131		29	29	73

The review identified logos from thirty-six individual food and beverage companies and we classified most (n=25) as healthy, 10 we classified as manufacturing unhealthy food products, one was not classified as a full product list was not available. Seven of the 17 logos from individual food or beverage product brands we classified as unhealthy, four as healthy, and six were unclassified.

Fifteen food or beverage brands/companies sponsored more than one sport (see Table 2). Four sponsored both NSOs and RSOs. Eight of the 15 we classified as unhealthy brands or companies. New World Supermarket’s logo appeared on 15 websites, sponsoring more sports than any other company, the majority being RSOs. Other frequent sponsors included Pak n’ Save supermarkets (n=9), McDonalds (n=9) and Coca Cola (n=8).

**Table 2:** Food and beverage brands/companies sponsoring more than one NSO/RSO

**Sponsor**	**Brand type/company**	**Total sponsorships**	**National sporting organisations**	**Regional sporting organisations**	**Sports sponsored**
		**n =**	**n =**	**n =**	**n =**
**New World**	Supermarket	15	1	14	6
**Pak n’ Save**	Supermarket	9	0	9	4
**McDonalds**	Quick service restaurant	9	2	7	4
**Coca Cola**	Beverage company	8	1	7	4
**Anchor**	Dairy company	6	0	6	1
**Mad Butcher**	Butcher	4	0	4	3
**Subway**	Quick service restaurant	4	0	4	4
**Milo**	Beverage	3	1	2	1
**Powerade**	Beverage	3	0	3	2
**Mizone**	Beverage	2	2	0	2
**Heavens Bakery**	Bakery	2	0	2	2
**Hubbards**	Cereal company	2	0	2	2
**Eta**	Snack food company	2	0	2	2
**Cadbury**	Confectionery company	2	0	2	2
**Fresh Choices**	Supermarket	2	0	2	2

Of the total of 186 logos that appeared on the websites, logos of bars and restaurants (n=63) appeared most often, followed by logos of unhealthy brands or companies (n=52) and healthy brands/companies (n=38). Most of the 33 unclassified logos belonged to supermarkets.

Rugby had more food and beverage sponsors than other sports. The majority of these were restaurants and bar. See Table 3 for results of frequency of sponsor’s logos by type. Most food and beverage sponsors in rugby league and cricket were classified as healthy. Only two sports, touch rugby and badminton, had more unhealthy than healthy sponsors; however these sports had few sponsors overall.

**Table 3:** **Frequency of food and beverage sponsors**’ logos by sport and sponsor type*

**Sport**	**Total websites n**	**Websites with sponsors’ logos n**	**Unhealthy foods n (%)**	**Healthy foods n (%)**	**Unclassified n (%)**
**Rugby**	27	26	23 (25.0)	21 (22.8)	48 (52.2)
**Other**	47	22	2 (22.2)	1 (11.1)	7 (66.7)
**Hockey**	27	20	1 (20.0)	0 (0)	4 (80.0)
**Basketball**	21	15	4 (26.7)	4 (26.7)	7 (46.6)
**Netball**	13	13	1 (11.1)	0 (0)	8 (88.9)
**Tennis**	17	12	2 (33.3)	1 (33.3)	2 (33.4)
**Golf**	14	11	4 (40.0)	0(0)	6 (60.0)
**Touch rugby**	13	11	4 (57.2)	0 (0)	3 (42.6)
**Squash**	11	10	1 (20.0)	2 (40.0)	2 (40.0)
**Cricket**	7	7	4 (28.6)	5 (35.8)	5 (35.6)
**Football**	8	7	1 (33.3)	0 (0)	2 (66.7)
**Rugby league**	5	4	1 (20.0)	4 (80.0)	0 (0
**Badminton**	4	3	4 (100)	0 (0)	0 (0)
**Water Ski Racing**	2	1	0 (0)	0 (0)	2 (100)
**Total**	216	162	52	38	96

* Other includes sports with only one food and beverage sponsor.

### Key informant interviews

Informants considered food and beverage brands and companies in NZ undertook limited sports marketing and confined their activities to sponsorships with a few high profile sports. Participants thought these sports had a strong competitive advantage as their regular television coverage helped raise their profile, provided exposure for sponsors, and ultimately helped the sport attract more sponsorship. As one informant noted;

" because we don’t get television coverage there’s not a lot of demand for companies coming in wanting to advertise. It’s slightly different to a rugby, or a soccer [football] or a netball court where there’s lots of television. And a lot of the sponsorship comes back to what sort of coverage they’re getting in the media on television. We don’t get a lot. "

Informants identified six examples where food and beverage companies supported their sponsorship investment in NZ sports with complementary marketing. Four sponsorships established by the NSO flowed to the RSO and clubs, three of these targeted children; of these, two were classified as unhealthy. These sponsorships included the national junior cricket programme, national sponsorship of netball, and sponsorship of ‘player of the day’ certificates for junior football and touch rugby. ‘Player of the day’ certificates are distributed each week at the conclusion of a game to acknowledge the team’s best player. The award typically includes a voucher for a food item from a sponsor and a branded certificate.

All complementary marketing provided significant brand exposure for sponsors and utilised several techniques to encourage purchase. For example, national sponsorship of an RSO managed junior cricket skills programme provided the sponsor with naming rights and opportunities to distribute branded giveaways (such as caps and cricket balls), product samples, and discount vouchers to participants. National netball sponsorship gave the sponsor naming rights to the national team, national age group representative teams, and international home test series. The sponsor also provided spectator prizes, catering for volunteers, and vouchers for volunteers’ awards, which were distributed on a weekly basis to netball centres nationally. Sponsorship of ‘player of the day’ certificates allowed a fast food chain to provide product vouchers to children playing football and touch rugby at club level each week. A licensed promotion enabled one sponsor to use rugby players’ names and images on collector cards given to purchasers as a reward that encouraged repeat purchase (necessary to obtain a full set of cards).

All informants identified the main benefit of sponsorship as financial. The commercial investment enabled them to allocate more resources to their sport. One informant stated; “*clearly it puts more resources into the sport, number one and that’s the key benefit for us. We can do what we want to do easier.”* Sponsorship income was usually incorporated into general funds that supported elite teams including: travel and accommodation for teams, officials and referees; and administration expenses. Informants also saw these arrangements as providing opportunities to promote their sport through the associated advertising featuring their players and teams.

Informants described how sports administrators reviewed potential sponsorships, considering the appropriateness of the sponsor, and any potential negative impacts an association might have. Administrators reviewed potential sponsors carefully to ensure the brand, and associated messages, aligned with their sport. They appeared to believe a good ‘fit’ benefitted their sport as well as the sponsor.

A few informants noted that, after internal debate, their organisations had declined quick service restaurant company sponsorship as senior administrators did not consider the relationship appropriate. One informant explained;

a fast food company came to us and offered some reasonable money and it was quite a good intellectual discussion, does it sit with our brand and what would this do to the brand when you’re about healthy lifestyles, and all these other things when suddenly you attach a fast food which has all these other connotations attached to it.

However, other informants indicated that any benefits of being associated with a healthy food sponsor (or the drawbacks of being involved with an unhealthy sponsor) were weighted less than the funding resulting from sponsorship. One informant noted;

I guess that we were looking for healthy foods, healthy sponsors, whatever that might look like. Now, that said, I’d be the first to say if somebody came to us with food that wasn’t quite healthy but had a big cheque book, I’d probably look at the cheque book in preference.

## Discussion

Interviews with key informants suggested food and beverage sponsorship was not extensive in NZ sport. Data from reviews of NSO/RSO websites supported this, finding more than three quarters (75.9%) had no overt links with food or beverage companies. The majority of food and beverage companies sponsoring NSO/RSOs were unclassified including bars, restaurants, and supermarkets, with the balance of sponsors fairly evenly split between brands and food companies classified as healthy or unhealthy.

Informants identified six examples where food and beverage companies supported their sponsorship by integrating it with other marketing activities. These included national sponsorships of netball, rugby, and junior cricket, provision of player of the day certificates for junior touch rugby and junior football, and licensed promotions with rugby. Four of these sponsorships targeted children and two promoted foods or beverages classified as unhealthy.

Food and beverage companies sponsoring the national teams of popular NZ sports used operant and respondent conditioning to support their sponsorships [12]. They also drew on vicarious learning which promotes new behaviour by using role models. Sponsorships supported by advertising linked the players’ athleticism and high performance with consumption of the sponsors’ products and implied use of the featured brands could facilitate similar performance levels.

Analysis of logos featured on websites gives only a limited insight into sports sponsorship’s effects. High profile sports are likely to be regularly televised, thus creating repeat brand exposure, and supporting strong brand attribute associations. When sponsors established relationships with NSOs they gained access to regional clubs and youth players and provided them with product samples, branded merchandise and vouchers.

Only one third of the food and beverage products or companies sponsoring sport in NZ were classified as unhealthy which may reflect sporting organisations’ preference for a strong ‘fit’ over sponsorship income. Only a few cases allowed food companies to market unhealthy products directly to children through their associated clubs, with potential negative impacts on their junior athletes’ diets.

This study addresses some potential limitations of earlier research [10,11] that used logo counting to assess sporting organisations’ use of unhealthy sponsorship. Counting logos does not capture the extent of food and beverage marketing in sports settings and may underestimate the extent of sponsorship. The mixed methods approach adopted in this study enabled identification of food and beverage companies sponsoring sport, and marketing strategies supporting sponsorships. This study provides a better estimation of the nature and extent of food and beverage marketing. However, this study did not identify food and beverage sponsorship in sports clubs and further research is required to examine this question.

Companies and products in this study were classified as healthy or unhealthy using the NZF&B system. This approach proved effective for classifying individual foods but many sponsors were food companies with diverse product ranges that we classified according to the majority of foods manufactured. Analysing company sales data would provide a more accurate picture in future research. A further limitation of this study is the large group of unclassified sponsors, mostly bars and restaurants. Classification may have been strengthened by adopting a Delphi survey process as used in Australian studies [9,10]. Criteria in these surveys include companies selling alcohol [9,10].

This study did not collect data on sponsorship of individual athletes, or franchised NZ teams participating in trans-Tasman competitions and international broadcast sport. These are all areas for further research.

## Conclusions

While there is limited food and beverage sponsorship of New Zealand sport, both healthy and unhealthy food and beverage brands and companies do sponsor sport. A few support their sponsorships with marketing activities that directly encourage consumer purchase and maintain brand salience. These sponsorships of high profile, regularly televised sports provide repeat brand exposure which may support favourable brand attribute associations.

Behaviour modification theory suggests unhealthy food and beverage sponsorships may foster consumption of unhealthy food products and dilute recommendations promoting healthy eating in sports settings. Sports codes and policy makers could encourage the adoption of healthier food choices. For example they could require all members to consider the wider implications of their sponsorship affiliations and could place a higher priority on health than revenue. National policy could assist sporting bodies by replacing unhealthy food sponsorship with healthy sponsorship. Effective models for this latter approach already exist, given successful replacement of tobacco sponsorship with health sponsorship following the introduction of the 1990 New Zealand Smoke-free Environments Act.

This NZ case study found food and beverage company sponsorship supported by marketing activity was associated with popular, televised sports. NZ provides a good opportunity to study food and beverage sponsorship of sport because of the popularity of sport and the high participation rates. However, NZ is a small country with popular sports different to those in other countries. Nevertheless, given the global nature of the food industry, these findings may be relevant elsewhere.

## Abbreviations

IMC: Integrated Marketing Communications; NSOs: National sporting organisations; NSO/RSOs: National and regional sporting organisations; NZF&B: NZ Food and Beverage Classification System for Years 1–13; RSOs: Regional sporting organisations.

## Competing interests

The authors declare they have no competing interests.

## Authors’ contributions

MC carried out the website reviews, conducted and analysed key informant interviews and drafted the manuscript. LS conceived of the study, participated in the design of the study and helped to draft the manuscript. JH participated in the design of the study and helped to draft the manuscript. RE participated in the design of the study, advised on analysis of results, and helped to draft the manuscript. AM conceived of the study and helped to draft the manuscript. All authors read and approved the final manuscript.

## Pre-publication history

The pre-publication history for this paper can be accessed here:

http://www.biomedcentral.com/1471-2458/13/126/prepub
